# Penile calciphylaxis—a complicated case managed with circumcision and glansectomy

**DOI:** 10.1093/jscr/rjab590

**Published:** 2022-01-15

**Authors:** Ellen O’Beirn, Muheilan Muheilan, Rowan Casey

**Affiliations:** Department of Urology, Tallaght University Hospital, Dublin D24NR0A, Ireland; Department of Urology, Tallaght University Hospital, Dublin D24NR0A, Ireland; Department of Urology, Tallaght University Hospital, Dublin D24NR0A, Ireland

## Abstract

Penile calciphylaxis is a serious manifestation of calcifying uremic arteriolopathy, with only a small number of cases reported in the literature. It is rare, characterized by calcification within the walls of small vessels, resulting in ischaemic changes to the skin, and is mainly seen in patients with end-stage renal failure (ESRF). Management of penile calciphylaxis is difficult, with both conservative and surgical approaches advocated for. Due to their comorbidity profile, patients with penile calciphylaxis can present multiple management challenges. We present a case of penile calciphylaxis in a patient with ESRF who was initially managed conservatively, and then underwent circumcision and glansectomy due to a necrotic glans penis and non-resolving penile pain. The patient was spared a partial penectomy and went on to make a full recovery.

## INTRODUCTION

Calcifying uremic arteriolopathy (CUR) is a rare condition characterized by calcification within the walls of small vessels, resulting in ischaemic changes to the skin [1]. It is very commonly seen in patients with end-stage renal failure (ESRF), especially those on dialysis [2, 3]. Penile calciphylaxis is an unusual presentation, with various management options employed based on the literature. Patients most commonly present with a painful ischaemic penile lesion. Predisposing factors include ESRF, dialysis, obesity and diabetes. It is often associated with a high mortality, especially when super-imposed infection is present.

## CASE REPORT

A 41-year-old male presented to the emergency department with penile pain for 1 week, associated with foreskin bleeding, penoscrotal swelling and shortness of breath. He had a history of type 1 diabetes mellitus, hypertension, peripheral arterial disease (PAD), single toe amputation and ESRF secondary to diabetic nephropathy for which he was on haemodialysis. On examination he was alert, haemodynamically stable, afebrile with generalized lower limb oedema. External genitalia examination revealed bilateral scrotal swelling, foreskin erythema and a foul smell. On palpation, the foreskin was tender, nodular. The foreskin was not retractable due to long standing phimosis and the meatus could not be identified.

The bloods showed raised leukocytes at 12400 × 10^9^/L, C-reactive protein of 24 mg/L, estimated glomerular filtration rate of 22 ml/min/1.73m^2^, creatinine of 472 umol/L, normal blood glucose, corrected calcium of 2.3 mmol/L, parathyroid hormone of 218 pg/ml and an albumin 34. Blood cultures came back negative. Computed tomography (CT) of his abdomen and pelvis showed bilateral inguinal lymphadenopathy of uncertain cause.

**Figure 1:**
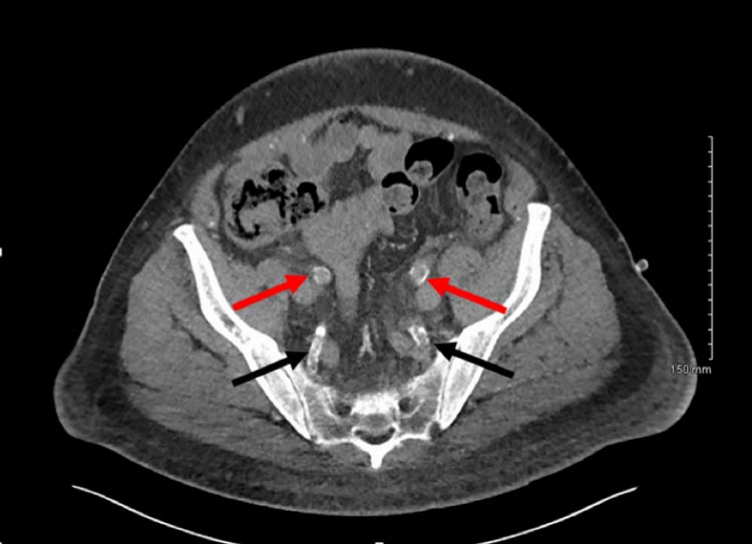
Axial slice of a non-contrast CT AP with red arrows showing calcified external iliac arteries and black arrows showing calcified internal iliac arteries.

Surgical treatment consisted of a dorsal slit, biopsy of glans and foreskin and urethral catheter insertion under general anaesthetic. Intra-operatively the glans had an appearance of patchy necrosis (Fig. 1). Histopathology results showed inflammation with no evidence of malignancy, consistent with an ischaemic process.

**Figure 2:**
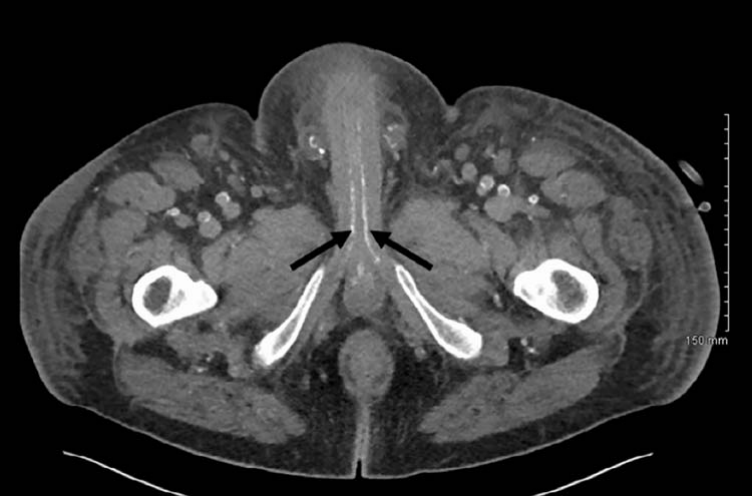
Axial slice of a non-contrast CT AP with arrows delineating calcified cavernosal arteries.

**Figure 3:**
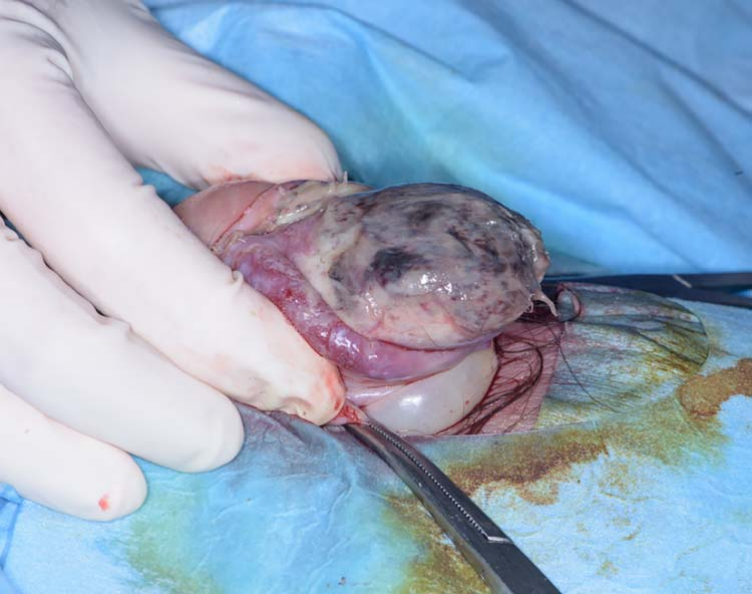
Intra-operative photo after dorsal slit procedure showing ischaemic patches on the glans of the penis.

Post-operatively the patient was experiencing ongoing severe penile, examination of the glans was not possible due to oedema and pain. Our patient underwent dialysis to offload excess fluid. A CT angiogram revealed extensive peripheral vascular arterial calcification, including calcification of the internal pudendal and cavernosal arteries (Figs 2 and 3). The inguinal lymphadenopathy was shown to be reactive.

One week post-operatively a re-look examination under anaesthaesia was performed. At flexible cystoscopy the distal urethra had a red dusky appearance, but otherwise normal. The glans remained necrotic and a circumcision and glansectomy was performed. The histopathology demonstrated necrosis and vessel calcification in keeping with calciphylaxis. The patient’s pain dramatically improved post-procedure, and bedside dressing changes were carried out three times per week under a penile block. Vascular review did not recommend any role for endovascular stenting, angioplasty or vasodilator medication. Three weeks post-operatively, he was discharged home. Follow-up in the urology outpatient department 2 weeks later showed resolution of the oedema and healthy granulation tissue of the distal penile skin. Penile pain had resolved.

## DISCUSSION

Calciphylaxis is a rare condition caused by calcification of small vessels, resulting in occlusion, ulceration and necrosis of overlying subcutis and skin [2]. It mainly effects patients on dialysis for ESRF, with a prevalence of 1–4% [3, 4]. Penile calciphylaxis has a reported incidence rate of 6% [5].

Female gender, diabetes mellitus and hyperphosphataemia have been identified as strong risk factors. ESRF, duration of dialysis, Caucasian ethnicity, obesity, hypoalbuminaemia, warfarin and autoimmune conditions are other well reported risk factors [4, 6]. Our patient was on haemodialysis secondary to diabetic nephropathy, as well as being hypertensive and obese. His albumin was at the lower level of normal, which is another possible contributor to his calciphylaxis.

Similar conditions include diabetic vascular disease, purpura fulminans, atheroembolic disease, antiphospholipid syndrome, PAD, vasculitis and necrotizing infection. Biopsy would be the definitive investigation to differentiate between these conditions [1].

The exact pathogenesis of calciphylaxis remains unclear [7]. Histopathologically, calcium deposition is seen in the media of small vessels, causing intimal thickening and luminal narrowing [8].

Calciphylaxis can present as painful skin lesions, super-imposed infection, ulceration and black eschar formation. Skin lesions in the buttocks and thighs are most commonly seen. Rarely, ischaemia of vascular supply to the muscle, brain, lung and gastrointestinal (GI) tract has also been reported [1, 6]. Penile calciphylaxis is a rare presentation of systemic calciphylaxis, with our case presenting similarly to other reported cases, with penile pain, however initially no necrosis was evident in our case due to the patient’s phimosis [1, 5, 9–12]. On performing a dorsal slit in theatre, patchy necrosis of the glans penis became apparent (Fig. 1).

The gold standard for the diagnosis remains skin biopsy. Biopsy of the foreskin at the time of dorsal slit did not elucidate any calciphylaxis, however did out rule malignancy. Glansectomy and circumcision confirmed calciphylaxis with vessel calcification and necrosis. However, often times diagnosis is made based on presentation, bloods and imaging, due to the risk of biopsy potentiating infection. Radiological investigations can aid diagnosis. CT is the most sensitive, assessing the extent of vascular calcification, as in our case [1] (Figs 2 and 3).

Treatment is controversial, with no unanimous management plan in the literature. This cohort is often very comorbid, as a result in less severe cases conservative management may be undertaken. This involves local wound debridement and analgaesia. Some treatment success has been shown with hyperbaric oxygen therapy in promoting tissue healing and sodium thiosulphate as an anti-chelating agent, however the evidence supporting this is lacking [13]. In a review of 34 cases managed conservatively the majority of them died, compared to 25% who underwent early surgery who died, suggesting that surgical intervention may be superior [14]. As in our case, circumcision and glansectomy treated this patient, and spared him from a partial penectomy, as is often performed in these cases.

In conclusion, penile calciphylaxis represents a rare and serious condition, that must prompt immediate attention. Early surgical intervention should be considered as this can often be a fatal diagnosis. Our patient was successfully treated with circumcisions and glansectomy, which may warrant consideration for more conservative surgical approaches than partial penectomy in the treatment of penile calciphylaxis.
